# Face located skin anthrax

**DOI:** 10.1590/0037-8682-0044-2022

**Published:** 2022-08-05

**Authors:** Fatma Kesmez Can

**Affiliations:** 1 Atatürk University School of Medicine, Department of Infectious Diseases and Clinical Microbiology, Erzurum, Turkey.

**FIGURE 1:**
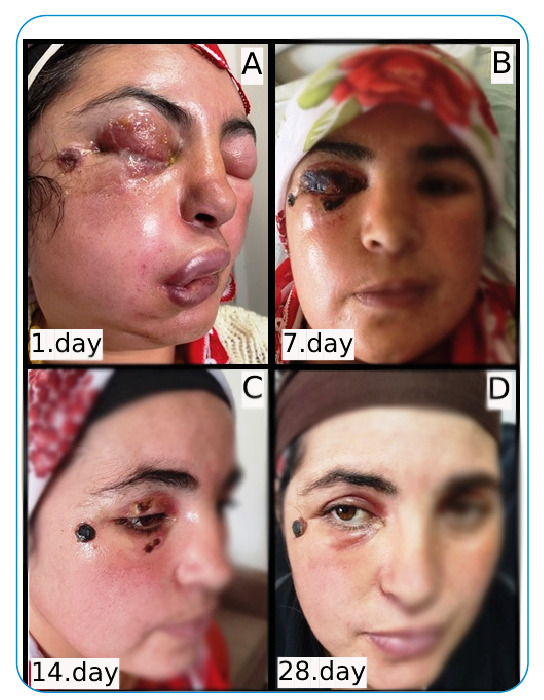
1-28-day follow-up of a patient with anthrax.

A 34-year-old woman was admitted to the Atatürk University Hospital due to complaints of facial swelling, fever, and mild shortness of breath. The patient was engaged in livestock farming, had no additional disease, and slaughtered animals 8 days ago. She reported that a pimple-like scar appeared on the side of her eye 5 days ago, and her face swelled. She only received three doses of amoxycillin-clavulanic acid. Her general condition was moderate, with a body temperature of 38°C. She had edema, erythema in the bilateral periorbital region, and a 1×1 cm black ulcerated lesion in the outer lateral aspect of the right eye (Figure 1A); gram-positive bacilli were observed in the Gram stain of the sample taken from the lesion area of the patient, who was thought to have anthrax. Reverse transcription polymerase chain reaction assay of the lesion sample showed presence of *Bacillus anthracis*, but bacterial reproduction was not observed in the culture^1^. As the patient had intense edema, crystalline penicillin (24 min/day) and methylprednisolone (80 mg/day) were administered. During follow-ups, the edema on his face decreased, while a black eschar tissue appeared on the right eyelid and ulcerated lesion. Steroid treatment was completed within 7 days (Figure 1B). Antibiotic therapy was administered for 14 days; afterward, the patient was discharged (Figure 1C). The patient returned to the hosrpA checkup was performed on the 14th day after discharge (Figure 1D).

In endemic areas, cutaneous anthrax can occur in different settlements. Hence, an early diagnosis should be made if the patient develops a typical painless skin lesion and a history of contact with infected animals or their products^2^ ^,^ ^3^.
